# Pharyngeal endoscopic submucosal dissection: detection, technique, and considerations for Western adoption

**DOI:** 10.1016/j.igie.2026.03.009

**Published:** 2026-03-27

**Authors:** Robert Bechara, Akiyoshi Ishiyama, Sasaki Toru, Ryosuke Kamiyama, Hiroki Mitani, Yukiko Sato, Toshiyuki Yoshio

**Affiliations:** 1 Division of Gastroenterology, Department of Medicine, Queen's University, Kingston, Ontario, Canada; 2 Department of Gastroenterology, Cancer Institute Hospital, Japanese Foundation for Cancer Research, Tokyo, Japan; 3 Department of Minimally Invasive Transoral Surgery, Cancer Institute Hospital, Japanese Foundation for Cancer Research, Tokyo, Japan; 4 Department of Head and Neck Oncology, Cancer Institute Hospital, Japanese Foundation for Cancer Research, Tokyo, Japan; 5 Department of Pathology, Cancer Institute Hospital, Japanese Foundation for Cancer Research, Tokyo, Japan

## Abstract

**Background and Aims:**

Pharyngeal squamous cell carcinoma (SCC) is increasingly detected at earlier stages in Japan, largely due to routine image-enhanced endoscopy and systematic pharyngeal examination. Endoscopic submucosal dissection (ESD) is now a preferred minimally invasive treatment option for superficial SCC, with excellent outcomes. Despite this, the technique remains underutilized in the West, even though the incidence of pharyngeal cancer continues to rise.

**Methods:**

This review brings together current evidence on pharyngeal ESD, indications, technique, outcomes, and adverse events.

**Results:**

Across contemporary series, en bloc and R0 resection rates are consistently high, with serious adverse events occurring infrequently. Nonetheless, pharyngeal ESD involves unique challenges stemming from complex anatomy, specialized anesthesia, and specific exposure techniques.

**Conclusions:**

We highlight practical barriers limiting adoption in the West, particularly limited knowledge in optical diagnosis, and propose targeted education to close the knowledge gap. As demand increases for minimally invasive, organ-preserving treatments, pharyngeal ESD is poised to have an important role for the treatment of early pharyngeal SCC in Western practice.

## Key points

• Pharyngeal endoscopic submucosal dissection is a safe, effective, and minimally invasive treatment for superficial squamous cell carcinoma with excellent long-term outcomes.
• Lesion detection and risk stratification rely on detailed endoscopic examination incorporating the Japan Esophageal Society magnifying endoscopic classification of intrapapillary capillary loops.
• Laryngeal edema is the most clinically relevant adverse event; it is usually mild and managed conservatively, but can occasionally require airway intervention.
• Unique technical challenges in pharyngeal endoscopic submucosal dissection include working in a challenging anatomical location, requirement of specialized exposure methods, and specialized anesthesia requirements.
• A major barrier to Western adoption is the low rate of early lesion detection, largely due to limited training in optical diagnosis and routine pharyngeal examination.

## Introduction

Pharyngeal squamous cell carcinoma (SCC) is a challenging cancer that often presents at advanced stages with poor outcomes. Early detection of superficial pharyngeal neoplasia has improved in Japan because of routine screening in high-risk patients, leading to increased identification of lesions amenable to endoscopic treatment.^1^^,^^2^

Endoscopic submucosal dissection (ESD) for pharyngeal SCC was first described in the English literature in 2006, to our knowledge, and has since become increasingly performed in expert centers across Japan.^3^ Despite consistently favorable oncologic outcomes, pharyngeal ESD remains almost exclusively performed in Japan, with only a few studies from Korea and China, and virtually none from Western countries.^4^^,^^5^ Limited adoption of pharyngeal ESD in the West is likely due to multiple factors, including the lack of systematic oropharyngeal examination during routine endoscopy, limited expertise in optical diagnosis, and the absence of screening programs for high-risk patients.

In Japan, the incidence of oral and pharyngeal carcinoma is approximately 5 per 100,000 and continues to rise.^1^^,^^6^ However, detection of superficial pharyngeal carcinoma has improved through systematic examination of the pharynx during routine upper endoscopy, widespread use of image-enhanced endoscopy, and integration of optical diagnosis into endoscopic training.^7^ In Japan, superficial pharyngeal carcinoma is frequently detected synchronously with esophageal SCC (ESCC) or during surveillance of patients with a prior history of ESCC, representing an important pathway for early detection. Although ESCC is less prevalent in Western populations, patients with current or prior ESCC remain a clearly defined high-risk group in whom targeted pharyngeal surveillance is appropriate. Importantly, this risk-based approach is distinct from routine pharyngeal examination during upper endoscopy, which can be performed opportunistically in all patients regardless of indication and has been shown to substantially improve detection of early pharyngeal neoplasia.^2^^,^^8^, ^9^, ^10^

In contrast, in the United States, the incidence is approximately 7.5 per 100,000 and has increased by about 1% annually since 2013, with the majority of cases diagnosed at advanced stages.^1^^,^^11^ In Western populations, many pharyngeal cancers are human papilloma virus (HPV)-driven oropharyngeal carcinomas, which are generally managed with surgery or chemoradiation. Nonetheless, ESD remains relevant for the subset of superficial, mucosal squamous lesions that are HPV-negative, although these may be less common than in Japan.^1^

Major risk factors for pharyngeal SCC include smoking, alcohol, and HPV infection.^12^ In addition, drinkers who experience a flushing reaction to alcohol and carry the aldehyde dehydrogenase 2 1/2 genotype have a 4-fold increased risk of developing pharyngeal cancer compared to those without this genotype.^2^ A personal history of ESCC is also a significant risk factor, with surveillance studies demonstrating incidence of metachronous cancers exceeding 17% of patients at 7 years.^8^

In Japan, pharyngeal ESD has become one of the preferred transoral treatment options for superficial pharyngeal SCCs. Alternative minimally invasive techniques, such as transoral robotic surgery (TORS), transoral videolaryngoscopic surgery (TOVS), and endoscopic laryngopharyngeal surgery (ELPS), may be selected based on tumor location and local expertise. In this review, we summarize the indications, technical aspects, outcomes, and adverse events (AEs) associated with pharyngeal ESD, and discuss considerations and strategies for integrating this technique into Western clinical practice.

## Methods

We conducted a structured narrative review of the literature examining pharyngeal ESD, focusing on indications, clinical outcomes, AEs, and long-term oncologic results. PubMed, EMBASE, and manual reference searches were performed to identify relevant studies published up to May 2025. Priority was given to multicenter prospective series and meta-analyses; smaller retrospective studies were included when higher-level evidence was unavailable. Certainty of evidence was evaluated using a modified Grading of Recommendations, Assessment, Development, and Evaluation (GRADE) approach adapted for narrative synthesis, assigning certainty levels (high, moderate, low) based on study design, sample size, consistency of findings, risk of bias, and length of follow-up (≥3 years). Using this schema, we rated large multicenter cohort studies with consistent results high certainty, whereas smaller or less-consistent studies were graded moderate or low certainty.^13^ Because of variability in reporting among contemporary series, some outcomes were not uniformly available. In cases of missing data (eg, recurrence rates, lymph node metastasis [LNM], specific AEs), available results were pooled independently for each outcome. Formal imputation was not performed. Where evidence was limited, we offer expert opinion based on extensive clinical experience. Our center (Cancer Institute Hospital, Japanese Foundation for Cancer Research, Tokyo, Japan) is among the highest-volume centers globally for pharyngeal ESD, performing over 100 cases annually, with cumulative experience now exceeding 1000 cases.

## Indications and risk stratification

The pharyngeal wall consists of 3 main layers: the epithelium, which lines the surface; the subepithelial (SEP) layer, which includes connective tissue; and the muscularis propria, composed of skeletal muscle. A key distinction from the esophagus is that it lacks a muscularis mucosae. For the purposes of ESD, the hypopharynx is typically divided into 4 subregions: the right and left piriform sinuses, postcricoid area, and posterior walls. Although formal guidelines are lacking, current literature consistently supports the following indications for pharyngeal ESD:

1. High-grade intraepithelial neoplasia, carcinoma in situ (CIS), or SEP carcinoma.
2. No evidence of muscular layer invasion on preoperative assessment, based on endoscopic evaluation (high-definition white-light and image-enhanced endoscopy) and cross-sectional imaging.
3. No evidence of lymph node or distant metastasis on preoperative imaging.
4. Lesions generally confined to no more than 2 contiguous subregions of the hypopharynx; extensive circumferential postcricoid or bilateral piriform sinus involvement is relatively contraindicated because of higher risks of deformation, aspiration, and stenosis.

These indications are based on multiple large cohort studies demonstrating a low risk of LNM in superficial pharyngeal cancers (Table 1).^4^^,^^14^, ^15^, ^16^, ^17^, ^18^, ^19^, ^20^, ^21^, ^22^, ^23^, ^24^, ^25^, ^26^, ^27^ The largest of these studies was a multicenter analysis by Katada et al,^24^ which included 662 lesions, of which 380 (57.4%) were CIS and 282 (42.6%) were SEP carcinomas. For CIS, the LNM rate was 0%, with a 3-year cause-specific survival of 100%. For SEP carcinomas, the LNM rate was 4.6%, with a 3-year cause-specific survival rate of 99.6%. Patients who developed LNM during follow-up were treated with neck dissection, radiotherapy, or chemoradiotherapy. Similar findings were reported by Fujii et al^28^ in their study of 633 lesions, confirming the 0% LNM rate for CIS, and 4.4% for SEP carcinomas.

**Table 1:** LNM risk based on invasion depth^4^^,^^14^, ^15^, ^16^, ^17^, ^18^, ^19^, ^20^, ^21^, ^22^, ^23^, ^24^, ^25^, ^26^, ^27^

Depth category	LNM rate (%)	3-year cause-specific survival (%)∗	Certainty of evidence†
CIS	0	100	High
SEP <1000 μm	∼1-2	>99	Moderate
SEP ≥1000 μm	4-5	>99	Low-moderate
SEP + LVI, budding, infiltration	5-10	Variable	Low

*CIS*, Carcinoma in situ; *LNM*, lymph node metastasis; *LVI*, lymphovascular invasion; *SEP*, subepithelial.

∗ Studies reporting at least median 3-year cause-specific survival.

† Certainty graded using modified Grading of Recommendations, Assessment, Development, and Evaluation framework.^13^

**Figure 1:**
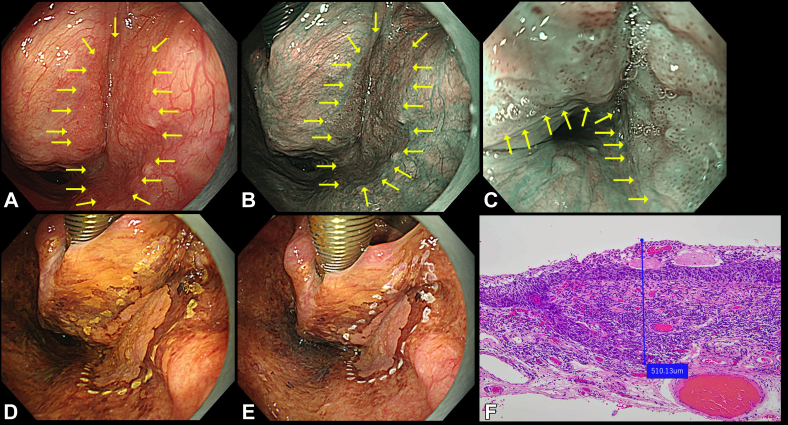
Superficial pharyngeal squamous cell carcinoma and measurement of subepithelial (SEP) squamous carcinoma. **A** and **B,** White-light and narrow-band imaging (NBI) views of a lesion in the right piriform sinus (*yellow arrows* delineate the lesion margins). **C,** Intrapapillary capillary loop changes are visible under NBI magnification (*yellow arrows* delineate the lesion margins). **D** and **E,** Lugol chromoendoscopy demonstrates the unstained area corresponding to the lesion. The pink color sign emerges after approximately 2 minutes. **F,** Histopathology (0.75% Lugol's) demonstrating measurement of SEP tumor thickness from the epithelial surface to the deepest point of carcinoma invasion.

Tumor thickness ≥1000 μm in SEP carcinomas has been identified in multiple studies as an independent predictor of LNM.^20^^,^^22^^,^^25^^,^^28^, ^29^, ^30^ Because of the absence of a muscularis mucosa in the oropharynx and hypopharynx, tumor thickness is measured from the surface of the carcinoma to its deepest point (Fig. 1). Tumor thickness ≥1000 μm is associated with a risk of LNM of 4-5%. Additional histologic features independently associated with LNM include lymphatic and vascular invasion, tumor budding, infiltrating growth pattern with indistinct boundaries, and positive deep resection margins.^27^^,^^28^

Recently, a “watch-and-wait approach” has been proposed for SEP carcinomas, including those with invasion depth greater than 1000 μm.^17^ In this approach, patients undergo close imaging surveillance, and if regional LNM develop, definitive treatment (neck dissection, radiotherapy, or chemoradiotherapy) is provided.^17^^,^^24^ However, the watch-and-wait approach requires strict adherence to high-frequency surveillance protocols with neck imaging (computed tomography [CT], magnetic resonance imaging, or ultrasound every 3 to 6 months) and shared decision-making. Although these series are limited, results suggest this conservative approach is suitable and is our current practice. In select cases of superficial pharyngeal carcinoma with cervical nodal involvement, transoral resection combined with neck dissection may be considered as a laryngeal-preserving strategy.

Ultimately, patient selection for pharyngeal ESD requires multidisciplinary input, balancing tumor features, patient comorbidities and preference, procedural expertise, and local resources. Alternative treatments may include TORS, TOVS, ELPS, radiation therapy, chemoradiation, or open surgery.

## Endoscopic examination and depth estimation

A structured and systematic pharyngeal examination during routine upper endoscopy has been shown to improve early detection of pharyngeal squamous neoplasia, particularly when applied in high-risk populations and when incorporated into standard endoscopic technique rather than as a formal screening program. During pharyngeal examination, patient phonation (eg, sustained vocalization such as “eee”) can be used as an adjunct to improve visualization of the piriform sinuses by dynamically altering pharyngeal and laryngeal anatomy. Although not required in all patients, phonation may be particularly helpful in high-risk individuals or when visualization is suboptimal. Accurate endoscopic prediction of invasion depth is essential when evaluating candidates for pharyngeal ESD. Gross morphology assessed using the Paris classification correlates with risk of SEP cancer. In the pharynx, the Paris classification for superficial squamous neoplasia is applied according to standard definitions. Slightly elevated lesions (Paris 0-IIa) demonstrate low-profile elevation less than the height of an open biopsy forceps cup (approximately 1.2 mm), whereas protruding lesions (Paris 0-I) show more-prominent exophytic growth exceeding this level. These distinctions are based on qualitative endoscopic appearance rather than precise height measurements.^31^ Tateya et al^32^ analyzed 139 pharyngeal SCCs, demonstrating significantly increased risk of SEP carcinoma with elevated morphology: 14% for flat lesions (Paris 0-IIb), 54% for slightly elevated lesions (Paris 0-IIa), and 100% for protruding lesions (Paris 0-I; *P* < .0001). Flat (0-IIb) lesions were the most common (71%), emphasizing the challenges of detection using white-light endoscopy.

**Figure 2:**
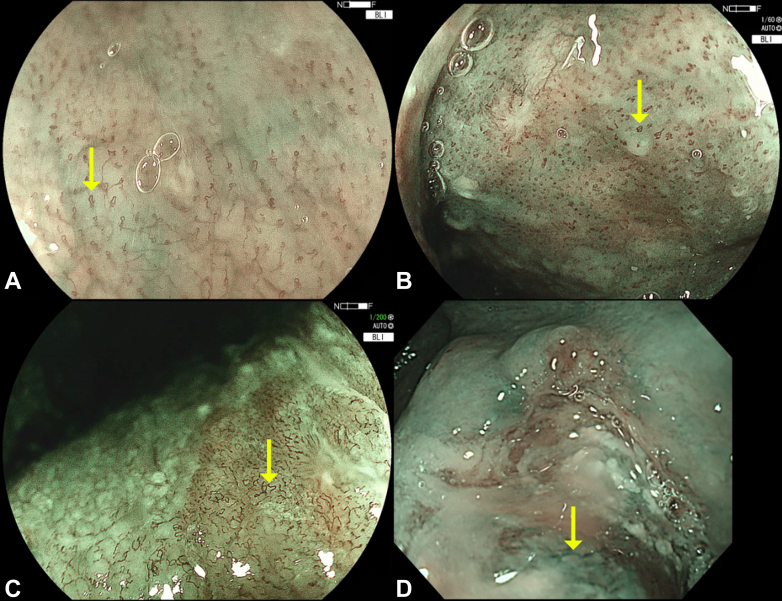
Representative intrapapillary capillary loop patterns according to the Japan Esophageal Society classification. *Yellow arrows* indicate the characteristic vessels: (**A**) type A, (**B**) type B1, (**C**) type B2, and (**D**) type B3.

Microvascular changes in pharyngeal SCC mirror those observed in ESCC. Characteristic alterations in intrapapillary capillary loops, from normal loops through progressive dilation, tortuosity, variations in caliber and shape to the formation of abnormal tumor vessels, are classified according to the Japan Esophageal Society as types A, B1, B2, and B3 (Fig. 2).^33^ These stereotypical microvascular changes improve lesion detection and diagnostic accuracy with the use of narrow-band imaging (NBI), which is superior to white-light endoscopy.^34^ Kikuchi et al^35^ demonstrated the frequency of SEP cancer was 20.3% for B1, 78.6% for B2, and 100% for B3 vessels (*P* < .05). Furthermore, tumor thickness increased with IPCL subtype: 650 μm for B1, 720 μm for B2, and 2257 μm for B3 vessels.

These findings were validated by Yamaguchi et al,^30^ who demonstrated that B2 and B3 vessels were independently associated with SEP invasion. Additionally, LNM rates strongly correlated with the IPCL subtype: 1.6% (1/63) for B1, 4.8% (2/42) for B2, and 55.6% (10/18) for B3 vessels (*P* < .001) Tumor thickness greater than 1000 μm and type B2 and B3 vessel patterns were independently predictive of lymphatic invasion and LNM. These data support magnifying NBI as a noninvasive tool for metastatic risk stratification. Thus, important distinctions exist between SEP invasion and tumor thickness as metrics in pharyngeal cancer. SEP cancers, by definition, extend beyond the epithelial layer. However, in the absence of a muscularis mucosa within the pharyngeal mucosa, consistent measurement of invasion depth is challenging and subject to interobserver variability.^27^ In contrast, tumor thickness, measured from the epithelial surface to the deepest point of invasion, has emerged as a more reproducible and clinically meaningful histologic parameter. Specifically, a tumor thickness of 1000 μm or greater more reliably predicts LNM and is currently considered the more robust prognostic marker compared to SEP depth.^25^

When biopsy is required in the pharyngeal region, it should be performed carefully to avoid significant bleeding and edema; use of small-capacity or pediatric biopsy forceps may be helpful to minimize tissue injury. When optical diagnosis is high confidence and endoscopic resection is planned, biopsy can be deferred because of definitive histology obtained after ESD.

Lugol's iodine chromoendoscopy is also valuable in assessing pharyngeal carcinoma, analogous to its role in ESCC. However, due to the risk of aspiration-induced chemical pneumonitis, Lugol's solution (0.75%-1.5%) should only be applied when the airway is protected with the patient under general anesthesia. Normal glycogen-rich squamous epithelium stains dark mahogany, and neoplastic or inflamed mucosa remains unstained (Lugol-voiding lesions), appearing yellow-orange (Fig. 1). Lugol-voiding lesions exhibit high sensitivity but limited specificity for squamous neoplasia. The pink color sign, observed 2 to 3 minutes after Lugol's application, provides both high sensitivity and specificity for carcinoma.^36^ In addition to its role in detection and characterization, Lugol's iodine is an important adjunct for delineating lesional borders and is routinely used. However, its superiority over NBI for this purpose has not been demonstrated.^37^ Although in our clinical experience some lesions may show equivocal delineation with either modality, the routine use of both NBI and Lugol's helps ensure optimal marking of the lesion prior to resection.

Despite these advanced imaging techniques, missed pharyngeal lesions remain problematic, with miss rates ranging from 35% to 85%.^38^^,^^39^ However, structured and systematic pharyngeal examination during routine upper endoscopy significantly improves early detection of pharyngeal squamous neoplasia.^9^

## Anatomy

**Figure 3:**
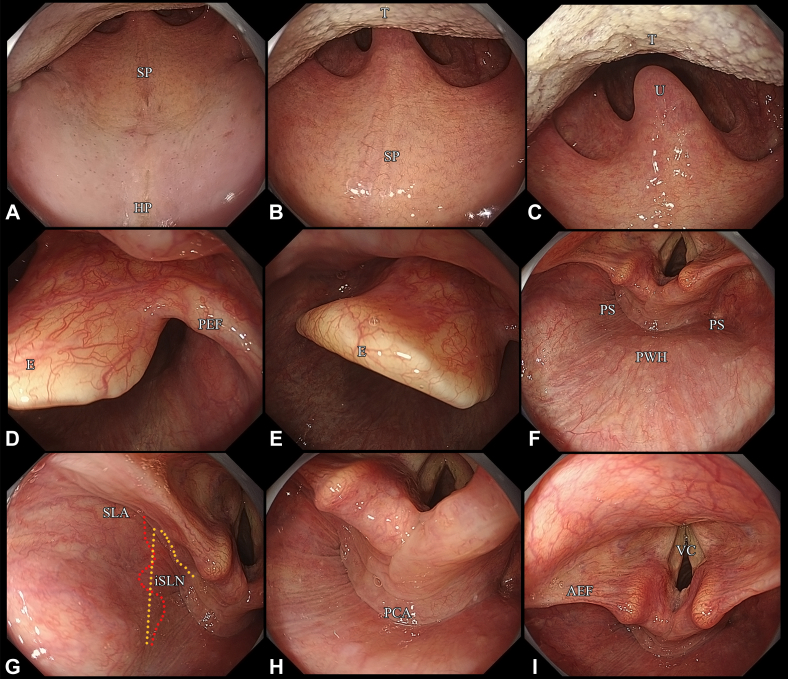
Key anatomical landmarks of the oropharynx, hypopharynx and larynx: (**A to C**) hard palate (HP), soft palate (SP), tongue (T), uvula (U), (**D to F**) epiglottis (E), pharyngoepiglottic folds (PEFs), posterior wall of hypopharynx (PWH), piriform sinuses (PSs), aryepiglottic folds (AEFs), (**G**) overlay of superior laryngeal artery (SLA), and internal branch of the superior laryngeal nerve (iSLN) (**H**) postcricoid area (PCA), (**I**) vocal cords (VCs) and aryepiglottic folds (AEF).

Successful pharyngeal ESD also requires familiarity with the complex anatomy of the pharynx. The oropharynx begins at the soft palate and extends to the pharyngoepiglottic folds (Fig. 3). Key anatomical landmarks include the soft palate, tonsils, vallecula, and base of the tongue. Distal to this, the hypopharynx extends from the pharyngoepiglottic folds to the upper esophageal sphincter. Notable areas in the hypopharynx include the posterior pharyngeal wall, piriform sinuses, and the postcricoid area. The hypopharynx then extends to the aryepiglottic folds, where it meets the larynx. Relevant areas of the larynx include the vocal cords, arytenoid cartilages, and the epiglottis. Beneath the mucosa of the piriform sinuses lie the internal branch of the superior laryngeal nerve (iSLN) and the superior laryngeal artery. Within the hypopharynx, laryngeal structures, such as the true vocal cords, false cords, and aryepiglottic folds, can also be examined. Approximately 80% of early pharyngeal SCCs arise in the hypopharynx, most commonly in the piriform sinuses, whereas the remaining 20% occur in the oropharynx.^10^ Resection of oropharyngeal cancers may extend to involve the epiglottis, and resection of hypopharyngeal cancers may extend to include the arytenoids and aryepiglottic folds.

## Technical aspects

Originally developed for early gastric neoplasia, ESD has since been applied to esophageal, colorectal, and duodenal neoplasia, and most recently to pharyngeal neoplasia. This progression demonstrates the shift of endoscopy from a primarily diagnostic modality to a minimally invasive therapeutic platform.

### Endoscopic equipment

#### Gastroscopes

Slim therapeutic gastroscopes (Olympus [Tokyo, Japan] GIF-H290T; Fujifilm [Tokyo, Japan] EG-840T; diameter 9.8 to 9.9 mm, channel 3.2 mm) are routinely used in pharyngeal ESD. An ultraslim gastroscope (Fujifilm EG-840TP; diameter 7.9 mm, channel 3.2 mm) may improve maneuverability in narrow oropharyngeal spaces; however, direct comparative outcome data remain lacking.^40^

#### Distal caps and knives

Transparent distal attachments provide stability, with the choice of cap design being operator dependent. Needle-type, insulated-tip, or multifunctional knives are all effective, and selection typically reflects operator preference.

### Specialized exposure and protective equipment

**Figure 4:**
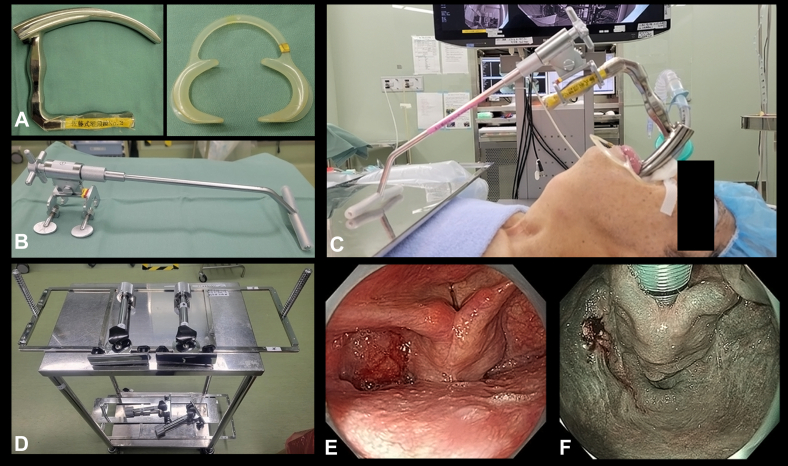
**A,** Curved laryngoscope and mouth opener. **B,** Laryngoscope holder/stand. **C,** Assembled curved laryngoscope system positioned in the patient. **D,** Equipment cart with laryngoscope holders and accessories. Endoscopic view of the hypopharynx before laryngoscope placement (**E**) and after placement (**F**), demonstrating improved exposure of the hypopharyngeal lumen and target lesion.

Pharyngeal ESD utilizes specialized equipment typically familiar to otolaryngologists but often novel to gastroenterologists (Fig. 4). A curved laryngoscope blade is positioned under direct endoscopic guidance, typically posterior to the epiglottis and above the vocal cords, and then secured using a holder and stand. Additional devices, such as mouth openers, palate retractors, and bite guards, ensure safe and optimal exposure, protecting oral structures and facilitating visualization.

### Anesthesia

General anesthesia is mandatory. Given that most early pharyngeal SCCs arise in the hypopharynx, particularly in the piriform sinuses, laryngeal elevation using a curved laryngoscope is required in most pharyngeal ESD procedures to achieve adequate exposure and optimize maneuverability. The anesthetic strategy varies according to lesion characteristics, procedural complexity, and institutional practices. Subglottic or nasotracheal approaches can eliminate the need for prophylactic tracheostomy. Elective tracheostomy may be considered for circumferential postcricoid lesions or for patients at high risk for severe laryngeal edema because of prior chemoradiation. The airway strategies, typical indications, and their respective advantages and limitations are summarized in Table 2.

**Table 2:** Airway management strategies

Airway strategy	Typical indications	Advantages	Limitations
Standard oral endotracheal tube (no elevation)	Very small, easily accessible oropharyngeal lesions	Simple, universally available	Limited exposure; seldom adequate for hypopharynx
Oral tube + laryngeal elevation (curved laryngoscope)	Most hypopharyngeal/piriform-sinus lesions	Excellent exposure	Requires ENT laryngoscope set-up
Subglottic airway (small-bore cricothyrotomy tube with balloon occlusion).^41^	Bulky lesions or high risk of postoperative edema	Optimal workspace; secures airway while eliminating oral tube obstructing movement	Minor neck incision; anaesthetist experience required
Nasal (nasotracheal) intubation^23^^,^^42^	Anterior epiglottis or tongue-base lesions	Removes oral tube obstruction, preserves laryngoscope space	Nasal trauma risk; limited if severe septal deviation
Tracheostomy^23^^,^^42^	Extensive aryepiglottic or supraglottic extension; rescue for severe edema	Definitive airway, maximal exposure	Invasive; reserved for elective high-risk cases or emergencies

*ENT*, Otolaryngology.

### Dissection and traction

#### Submucosal injection

To minimize the risk of laryngeal edema, pharyngeal ESD typically uses a minimal submucosal injection volume. Normal saline or viscous solutions mixed with a small amount of indigo carmine (± diluted epinephrine) are effective. In our practice, normal saline is preferred, and we recommend avoiding viscous agents because of their prolonged submucosal retention and the potential for increased risk of laryngeal edema. Although direct comparative studies are lacking, saline has the theoretical advantages because of rapid dissipation.^43^

#### Laryngeal contact

During marking and dissection, every scope pass contacting the larynx risks edema, particularly when the lesion spans the postcricoid or laryngeal surface. Operating at a slight distance from the lesion and avoiding unnecessary contact reduces mechanical trauma and potential risk for significant laryngeal edema. One way to minimize scope contact and trauma is the use of traction.

#### Traction techniques

Most traction techniques share similar principles but differ in their application. Initial procedural steps of circumferential incision and mucosal flap creation remain standard across traction techniques. Once completed, various traction techniques can be applied. Popular methods include the double-scope technique, clip-and-line/band method, curved forceps, and curved forceps with clip + band.^22^^,^^44^^,^^45^ The various traction techniques, along with their set-up, advantages, and limitations, are summarized in Table 3.

**Table 3:** Traction methods in pharyngeal endoscopic submucosal dissection

Traction method	Set-up	Advantages	Limitations
Double-scope (transnasal ultraslim)^22^	Treatment scope withdrawn; ultraslim scope inserted nasally, grasps flap, then parked at oropharynx while main scope re-enters	Dynamic, adjustable vector; excellent exposure; no accessory exchange. Does not require second tower.	Requires second scope and assistant, learning curve
Clip-and-line/clip-and-band^44^	Clip with nylon line or rubber band affixed to flap, tensioned externally	Simple, inexpensive, single operator	Fixed traction axis; may need redeployment
Curved forceps, ± clip and band^45^^,^^46^	Clip band placed; curved forceps introduced orally to grasp band. Curved forceps can also be used in isolation but limit endoscopic space	Strong multidirectional traction; useful in deep piriform sinus lesions	Requires ENT forceps; additional instrumentation. May limit endoscopic maneuverability

*ENT*, Otolaryngology.

Our current preferred traction method is the double-scope technique (Video 1, available online at www.igiejournal.org). In this approach, the treatment scope is first withdrawn, and an ultraslim gastroscope is introduced transnasally, equipped with grasping forceps to secure the mucosal flap. Once traction is applied, the ultraslim scope is withdrawn to the level of the oropharynx. The treatment scope is then reinserted, and ESD resumes with dynamic traction that can be adjusted in real time by manipulating the ultraslim scope and or the grasping forceps. This method provides excellent procedural flexibility and enhanced control during dissection.^22^

## Postprocedural management

Patients are typically extubated immediately postprocedure if laryngeal patency is confirmed by endoscopy and a positive cuff-leak test. For patients with confirmed severe edema or at high risk, temporary overnight intubation or prophylactic tracheostomy may be performed. Alternatives such as uncuffed cricothyrotomy with subglottic balloon occlusion may be used to avoid prolonged intubation or surgical tracheostomy.^19^^,^^41^^,^^47^

Postoperatively, patients are usually admitted for observation, with some institutions opting for initial intensive care unit monitoring. The use of prophylactic steroids and antibiotics is variable, with limited supporting evidence.^4^^,^^19^^,^^47^ Oral intake typically begins the day after the procedure, following satisfactory endoscopic assessment of laryngeal patency and edema, followed by clinical swallowing evaluation to assess aspiration risk. Swallowing function is assessed clinically, typically with bedside evaluation and, when indicated, formal assessment by speech language pathology. If significant edema is present, or other concerning features, oral intake is held until repeat endoscopic assessment is performed on days 3 and 4 to confirm resolution prior to initiating a diet.^4^^,^^19^^,^^43^ Patients are generally discharged within 4 to 7 days, once normal oral intake resumes and pain is adequately controlled with nonnarcotic analgesics.^22^^,^^40^^,^^48^

Long-term follow-up after pharyngeal ESD is essential to monitor for local recurrence, functional recovery, LNM, and metachronous lesions. Endoscopic assessments are completed every 3 to 6 months for at least 5 years, and in patients with SEP cancer, positive vertical margins, lymphovascular invasion, or other high-risk features, surveillance ultrasound, computed tomography/magnetic resonance imaging of neck ± chest is typically performed every 3 to 6 months.^19^^,^^22^

## AEs

Historically, AEs after pharyngeal ESD occur in approximately 10% of patients, with a majority related to airway proximity.^49^^,^^50^ In a meta-analysis by Kamal et al^49^ (2006-2020, 10 studies, n = 917 lesions), overall AEs occurred in about 10% of cases, with laryngeal edema being the most frequent at 11%.

In our pooled analysis from contemporary series (2021-2025, 10 studies, n = 889 lesions; Table 4), overall AE rates were lower at 4.6% (95% confidence interval [CI], 2.9%-7.0%), reflecting increased operator experience and technical refinement.^4^^,^^25^^,^^26^^,^^40^^,^^43^^,^^45^^,^^47^, ^51^, ^52^, ^53^ In these recent series, laryngeal edema of any grade occurred in 8.8% (95% CI, 6.0%-12.4%), and clinically significant edema requiring airway intervention was 2.0% (95% CI, 0.9%-3.8%). Aspiration events were reported in 1.6% (95% CI, 0.6%-3.0%), typically related to transient postoperative dysphagia, and were generally managed conservatively.^19^^,^^47^ Longer-term aspiration risk has been associated with pharyngeal deformation, with a deformation grade ≥2 linked to a 9% cumulative aspiration rate at 3 years.^53^ The long-term decline in swallowing function is not yet completely understood, and further investigation is warranted. Furthermore, injury to the internal branch of the superior laryngeal nerve can result in laryngeal sensory deficits and silent aspiration due to loss of the cough reflex; although the precise incidence of this AE in pharyngeal ESD remains undefined, it represents a significant clinical concern. Postoperative bleeding occurred in 1.8% of patients (95% CI, 0.9%-3.5%) and was managed endoscopically. Subcutaneous emphysema was rare (0.5%), generally asymptomatic, and resolved spontaneously.^4^^,^^19^ Fistulas and abscesses were infrequent (0.1%), with only 1 case of carotid rupture reported.^47^ Strictures or pharyngeal deformation occurred in 11.2% of patients (95% CI, 8.5%-14.4%), most often following extensive postcricoid or bilateral resections, and were typically treated successfully with endoscopic dilation.^54^ Overall, the AE rate of pharyngeal ESD is favorable, with serious AEs being rare.

**Table 4:** Adverse events and clinical outcomes (contemporary series, 2021-2025)^4^^,^^25^^,^^26^^,^^40^^,^^43^^,^^45^^,^^47^, ^51^, ^52^, ^53^

Adverse event	n (total)	Events	Incidence (95% CI)	Certainty of evidence∗	Comments
Overall AE‡	451	21	4.6% (2.9%-7.0%)	Moderate	Inconsistent reporting; most cases mild/self-limited
Laryngeal edema (severe requiring intervention)	451	9	2.0% (0.9%-3.8%)	Low	Rare, include emergent and prophylactic tracheostomy
Laryngeal edema (any)	340	30	8.8% (6.0%-12.4%)	Moderate	Mostly mild, self-limited edema
Postprocedural bleeding	486	9	1.8% (0.9%-3.5%)	Moderate	Managed endoscopically
Subcutaneous emphysema	403	2	0.5% (0.1%-1.9%)	Low	Mostly asymptomatic
Fistula/abscess (including carotid rupture)	734	1	0.1% (0.0%-0.8%)	Low	Single reported fatal event
Stricture/deformation	745	53	11.2% (8.5%-14.4%)	Low	Mostly postextensive resections
*Outcome*					
En bloc resection	817	804	98.4% (97.3%-99.2%)	High	Consistent across all cohorts
R0 resection	817	641	78.5% (75.5%-81.2%)	Moderate	Margin positivity largely related to deep margin involvement
Local recurrence	423	19	4.6% (2.7%-6.9%)	High	Rare events with long-term follow-up
Lymph node metastasis	754	36	4.8% (3.4%-6.5%)	Moderate	Primarily associated with SEP invasion and high-risk histology
3-5-year disease-specific survival	594	590	99.3% (98.3%-99.8%)	High	Excellent long-term oncologic outcomes

*AE*, Adverse event; *CI*, confidence interval; *SEP*, subepithelia.

∗ Certainty graded with modified Grading of Recommendations, Assessment, Development, and Evaluation framework.^13^†Median follow-up across included studies: 36 to 40 months.

‡ Overall AE rates were reported in fewer studies than category-specific events; denominators therefore differ and the overall AE rate should not be interpreted as the sum of individual categories.

## Clinical outcomes

In the systematic review by Kamal et al,^49^ the pooled en bloc and R0 resection rates were 94% (95% CI, 87%-97%) and 72% (95% CI, 62%-80%), respectively, with local recurrence and LNM observed in 1.9% (95% CI, 0.9%-4%) and 4% (95% CI, 2%-7%), respectively.^49^

In the contemporary data (2021-2025, 10 studies, n = 889 lesions; Table 4), clinical outcomes remain excellent.^4^^,^^25^^,^^26^^,^^40^^,^^43^^,^^45^^,^^47^, ^51^, ^52^, ^53^ The en bloc resection rate was 98.4% (95% CI, 97.3%-99.2%), with R0 resection achieved in 78.5% (95% CI, 75.5%-81.2%). Local recurrence was observed in 4.6% (95% CI, 2.7%-6.9%), and LNM occurred in 4.8% (95% CI, 3.4%-6.5%), with both events typically associated with tumor thickness ≥1000 μm or high-risk histologic features. Disease-specific survival at 3 to 5 years remains excellent at 99.3% (95% CI, 98.3%-99.8%), underscoring the durable oncologic efficacy of pharyngeal ESD.

## Barriers and future directions for Western practice

At present, robust comparative data directly comparing oncologic outcomes and AEs of pharyngeal ESD with transoral surgical approaches such as TORS or TOVS are lacking, with available evidence largely derived from single-modality retrospective series.^24^^,^^26^ In appropriately selected superficial disease, oncologic outcomes appear favorable across modalities, and treatment selection is therefore largely driven by lesion characteristics, anatomical location, and local expertise. Pharyngeal ESD is particularly well suited for superficial, flat, or slightly elevated lesions of the hypopharynx, especially within the piriform sinuses and posterior hypopharyngeal wall, where en bloc mucosal resection is feasible.^22^^,^^25^ In contrast, bulky or deeply exophytic lesions and those with extensive arytenoid or supraglottic involvement, may be better managed with transoral surgical approaches.^32^

Early lesion detection remains a major barrier to wider Western adoption of pharyngeal ESD.^38^^,^^39^ However, implementation of a brief systematic pharyngeal examination during routine upper endoscopy markedly improves lesion detection rates.^9^ Beyond detection and training, structural and interdisciplinary hurdles pose significant barriers to Western implementation. Unlike in Japan, where endoscopists frequently manage pharyngeal pathology, Western practice traditionally demarcates the pharynx as the domain of otolaryngology.

Consequently, gastroenterologists seeking to perform pharyngeal ESD may encounter credentialing challenges and medicolegal scrutiny regarding anatomical jurisdiction and airway management. Establishing a formal collaborative framework with otolaryngology, including protocols for intraoperative airway support and emergency surgical backup, is essential for institutional privileging and patient safety. Furthermore, technical feasibility relies on specific exposure devices, such as the rigid curved laryngoscope (Fig. 4A), which are standard in Japan but may lack widespread regulatory approval (eg, U.S. Food and Drug Administration clearance) or commercial availability in Western markets. Securing reliable supply chains for this specialized hardware is a prerequisite for establishing a functional program.

Credentialing for pharyngeal ESD in Western practice remains institution and jurisdiction specific, with no standardized pathway currently established. In practice, approval is typically achieved through multidisciplinary collaboration, most commonly involving otolaryngology and anesthesiology, given the need for specialized airway management and laryngeal exposure. Credentialing generally requires documented training and procedural experience, formal endorsement or coprivileging with otolaryngology, and agreed-upon protocols for intraoperative airway support and emergency surgical backup. These considerations underscore the need for institution-specific frameworks rather than a 1-size-fits-all credentialing model.

To advance Western practice, 3 core areas must be addressed:

1. **Training in pharyngeal ESD:** clinical training at high-volume Japanese centers with proctored procedures, followed by the establishment of programs in the West leading to dedicated Western training programs. Education should emphasize diagnosis, complex pharyngeal anatomy, specialized equipment, and strong interdisciplinary collaboration with otolaryngology.
2. **Screening and examination:** Routine integration of a structured pharyngeal examination into standard upper endoscopy, alongside development of targeted screening protocols for high-risk populations (eg, patients with prior ESCC, chronic alcohol use, or smoking history).
3. **Clinical data:** Creation of prospective Western registries to systematically monitor outcomes, maintain quality, and enable benchmarking against Japanese standards. Such registries will facilitate international collaboration and support development of global, evidence-based guidelines.

## Conclusions

Pharyngeal ESD has emerged as an elegant, organ-preserving, minimally invasive treatment for superficial pharyngeal SCCs. Although predominantly performed in Japan, interest and adoption internationally continue to grow. Like the global expansion of gastric, esophageal, colonic, and duodenal ESD, pharyngeal ESD is well positioned for the timely integration into Western clinical practice. Addressing current barriers through education, multidisciplinary collaboration, and international training will facilitate this transition, ultimately improving patient outcomes.

## Disclosure

The following author disclosed financial relationships: R. Bechara: Consultant for Olympus and Vantage Endoscopy. All other authors disclosed no financial relationships.

## Supplementary data

Video 1 Double Scope Pharyngeal ESD.
